# Simultaneous Reconstruction of Actuator and Sensor Faults in PMSG-Based Wind Turbines Under Various Anomaly Profiles: A Takagi–Sugeno Fuzzy Observer Approach

**DOI:** 10.3390/s26216905

**Published:** 2026-10-31

**Authors:** Nassima El Youssfi, Houcine Chafouk, Meriem Nachidi Labourel

**Affiliations:** 1 Univ Rouen Normandie, ESIGELEC, Normandie Univ, IRSEEM UR4353, 76000 Rouen, France; 2 ICAM, School of Engineering, Grand Paris Sud Campus, 77127 Lieusaint, France; 3 IBISC Laboratory, Université d’Évry Paris-Saclay, 40 rue du Pelvoux, 91080 Évry-Courcouronne, France

**Keywords:** wind energy conversion system, PMSG, fault estimation, Takagi–Sugeno fuzzy model, PI observer, unmeasurable premise variable, linear matrix inequalities

## Abstract

This paper addresses the simultaneous estimation of actuator and sensor faults in a permanent magnet synchronous generator (PMSG)-based wind energy conversion system (WECS) intended for isolated microgrids. The nonlinear dynamics of the wind turbine are represented by a Takagi–Sugeno (T–S) fuzzy model whose premise variable, the generator speed, is not measured; only the stator currents are measured. The faults, modeled as signals with bounded second derivatives, are treated as unknown inputs through an augmented state obtained by filtering the measured output, and an augmented fuzzy proportional–integral (PI) observer is adapted to this configuration. The structural conditions for reconstruction are stated and verified. The membership-function mismatch, which multiplies the true augmented state, is treated as a bounded input, whose bound is based on the differences between local matrices. Sufficient linear matrix inequality (LMI) conditions guarantee the uniform ultimate boundedness of the estimation error, with a prescribed decay rate and an $L_{2}$ attenuation level. The approach is evaluated in simulation, under a persistent measurement disturbance, for simultaneous actuator and sensor faults and then for biases, drifts and transients on each channel. The unmeasured speed is reconstructed with a root mean square error (RMSE) below 0.1% of its operating value and converges in about 2 s; the simultaneous faults are reconstructed with RMSEs of about 0.2% ($f_{a}$) and 3% ($f_{s}$) of their amplitudes, and the empirical $L_{2}$ gain, at most 0.92, remains below the attenuation level of 1.875 obtained from the LMI conditions.

## 1. Introduction

The transition toward sustainable energy systems highlights the essential role of renewable sources in achieving carbon neutrality and mitigating climate change [1,2]. In rural and remote areas, standalone microgrids offer a promising solution to ensure a reliable power supply and to support local infrastructure [3,4]. As these modern microgrids evolve, they are increasingly required to supply critical and sensitive loads, ranging from electrified farms to sustainable transport infrastructure, such as standalone green charging stations for electric vehicles [5]. Since these emerging applications demand high power availability and uninterrupted service, ensuring the reliability of the microgrid generation units is paramount.

PMSG-based wind energy conversion systems (WECSs) are widely used in microgrids for their high efficiency and power density [6,7]. However, harsh operating conditions challenge the reliability of wind turbines, since sensor and actuator faults can rapidly degrade performance and compromise microgrid stability [8,9]. Consequently, real-time fault detection and isolation (FDI) and fault-tolerant control (FTC) schemes are essential for safe and continuous operation.

Various fault diagnosis strategies have been proposed. Taouil et al. developed a modified sliding mode observer (SMO) for the pitch and torque systems [10]. Teng et al. proposed an adaptive observer-based FTC [11], while Bououden et al. presented a robust predictive control approach for time-delay systems [12]. T–S fuzzy model-based active fault-tolerant tracking control has also been studied for wind generators [13], as well as augmented observer schemes for the joint reconstruction of states and faults [14]. Stochastic filtering methods, such as the unscented (UKF) and extended (EKF) Kalman filters, have been successfully applied to the detection of actuator [15] and sensor [16,17] faults, respectively. For direct-drive PMSG-based wind turbines, faults in the machine-side and grid-side current sensors have been estimated by a sliding mode observer built on an augmented system obtained by filtering the measured outputs; the estimated errors are used to correct the measurements used by the controller, with simulation and experimental validation [18]. This approach was extended to the speed and dc-link voltage sensors [19]. For the speed sensor, an active fault-tolerant control scheme switching to a sensorless controller based on a sliding mode observer was validated experimentally [20]. These works consider sensor faults only.

However, the coupled aerodynamic and electromechanical dynamics of PMSGs are nonlinear. While Takagi–Sugeno (T–S) fuzzy modeling effectively represents such nonlinearities through local linear submodels [21,22], a major difficulty arises when the premise variable depends on a state that is not directly measured, leading to the so-called unmeasurable premise variable problem [23]. This is precisely the situation encountered here, since the generator speed used as the premise variable is not measured. For T–S models with unmeasurable premise variables, the simultaneous reconstruction of actuator and sensor faults has been addressed by a sliding mode observer with $H_{\infty }$ performance, the sensor fault being transformed into a fictitious actuator fault by output filtering [24]. Polytopic sliding mode observers have also been proposed for actuator fault estimation and fault-tolerant control of LPV descriptor systems with time delay [25]. In this context, most existing T–S observation and control schemes for PMSG-based wind turbines address either actuator faults alone [26] or state estimation without joint fault reconstruction [27]; the simultaneous estimation of actuator and sensor faults under external disturbances, with an unmeasurable premise variable, has received comparatively little attention for this class of machines.

Compared with the observers mentioned above, the T–S proportional–integral (PI) observer is adopted in this work for three reasons related to the problem addressed: it estimates the states and the faults simultaneously and directly reconstructs time-varying actuator and sensor faults, without a prior detection and isolation scheme [28]; it relies on the T–S fuzzy representation, which is suited to the nonlinearities of the PMSG and to the case of an unmeasurable premise variable; finally, its synthesis based on linear matrix inequalities (LMIs) includes a disturbance attenuation level.

This paper builds on the augmented fuzzy proportional–integral (PI) observer [28,29], which handles unmeasurable premise variables and time-varying faults through an LMI-based synthesis, and which was validated on a three-tank hydraulic system. Compared with that work, this paper:

- Formulates the observer for a PMSG-based wind turbine [26,27], with an actuator fault on the inverter voltage, a sensor fault on the current measurement and a measurement disturbance; the unmeasured rotational speed is the only premise variable, and the stator currents are the only measurements. The structural conditions of detectability and fault reconstructability are stated and verified for this configuration.
- Analyzes the estimation error with the membership-function mismatch treated as a bounded input acting on the error dynamics, following the $L_{2}$ approach of [30]; the mismatch is bounded through the differences between local matrices rather than through their norms, a prescribed decay rate is incorporated into the LMI conditions, and the uniform ultimate boundedness of the estimation error is established with an explicit bound, together with an $L_{2}$ attenuation level.
- Evaluates the observer using defined indices, namely the root mean square error (RMSE), the convergence time, the reconstruction delay and the empirical $L_{2}$ gain, under simultaneous actuator and sensor faults and under a persistent measurement disturbance.
- Reports the model and design parameters, including the numerical observer gains.

The remainder of this paper is organized as follows. Section 2 establishes the PMSG dynamics and the T–S fuzzy representation. Section 3 details the augmented system, the structural conditions, the PI-TS observer and the LMI-based gain synthesis. Section 4 discusses the simulation results, followed by the Conclusion in Section 5.

## 2. Modeling of the Wind Energy Conversion System

In this section, we present the mathematical model of a nonlinear PMSG-based WECS and its Takagi–Sugeno fuzzy representation.

### 2.1. Wind Turbine Modeling

The PMSG receives the aerodynamic torque produced by the wind turbine and converts it into electrical energy. The aerodynamic power captured from the wind and the tip-speed ratio λ(t) can be expressed as follows [26,27,31]:

(1) $$\left\{\begin{array}{cc} P_{aero}\left(t\right) & =0.5\;\nu \pi R^{2}V_{s}^{3}\;C_{P}\left(\lambda ,\beta \right)\\ \lambda (t) & =\frac{R\;\omega_{g}\left(t\right)}{V_{s}} \end{array}\right.$$

where ν, $V_{s}$ and R denote the air density, the wind speed and the blade radius, respectively. $C_{P}\left(\lambda ,\beta \right)$ is the power coefficient of the WECS, which depends on the pitch angle β and the tip-speed ratio λ(t). $\omega_{g}\left(t\right)$ is the mechanical rotational speed of the wind turbine. The analytical expression of the power coefficient is:

(2) $$C_{P}\left(\lambda ,\beta \right)=0.5\left[\frac{116}{\hat{\lambda }\left(\beta \right)}-0.4\beta -5\right]e^{-\frac{21}{\hat{\lambda }\left(\beta \right)}}$$

where

(3) $$\frac{1}{\hat{\lambda }\left(\beta \right)}=\frac{1}{\lambda +0.08\beta }-\frac{0.035}{1+\beta^{3}}$$

The turbine is assumed to operate at its optimal operating point under maximum power point tracking (MPPT), i.e., β=0, $\lambda =\lambda_{opt}$ and $C_{P}=C_{P_{\max}}$, so that $V_{s}=R\;\omega_{g}/\lambda_{opt}$. Substituting into (1), the mechanical torque $T_{M}=P_{aero}/\omega_{g}$ becomes

(4) $$T_{M}\left(t\right)=\frac{P_{aero}\left(t\right)}{\omega_{g}\left(t\right)}=T_{0}\;\omega_{g}^{2}\left(t\right),\;T_{0}=\frac{0.5\;\nu \;\pi \;C_{P_{\max}}\;R^{5}}{\lambda_{opt}^{3}}$$

### 2.2. PMSG Modeling

We consider the dynamic model of the PMSG in the rotating *d-q* reference frame [26,27]:

(5) $$\left\{\begin{array}{cc} L_{d}\frac{di_{d}\left(t\right)}{dt} & =\varpi_{e}\left(t\right)L_{q}i_{q}\left(t\right)-R_{s}i_{d}\left(t\right)+V_{d}\left(t\right)\\ L_{q}\frac{di_{q}\left(t\right)}{dt} & =-\varpi_{e}\left(t\right)L_{d}i_{d}\left(t\right)-\varpi_{e}\left(t\right)\phi_{mag}+V_{q}\left(t\right) \end{array}\right.$$

where $\varpi_{e}\left(t\right)=\rho_{n}\omega_{g}\left(t\right)$ is the electrical angular speed, $\rho_{n}$ is the number of pole pairs, and $\phi_{mag}$ is the permanent magnet flux. Assuming $L_{d}=L_{q}=L$, the electromagnetic torque is $T_{E}=1.5\rho_{n}\phi_{mag}i_{q}\left(t\right)$.

The mechanical dynamics are governed by:

(6) $$J\frac{d\omega_{g}\left(t\right)}{dt}+\Lambda \omega_{g}\left(t\right)=T_{E}-T_{M}$$

where Λ is the viscous friction coefficient and *J* is the moment of inertia, and where the motor sign convention of [26,27] is adopted, $T_{E}$ being the driving torque and $T_{M}$ the aerodynamic load torque.

The following modeling assumptions are adopted throughout this work. (i) *Drive train*: the PMSG is a direct-drive (gearless) wind turbine, so that the aerodynamic torque acts directly on the generator shaft. (ii) *Operating region*: the machine operates below rated wind speed at a pitch angle β=0 under MPPT, so that $\lambda =\lambda_{opt}$ and the aerodynamic torque reduces to $T_{0}\omega_{g}^{2}$; the wind speed is therefore implicit in the model, and the operating region is described by $\omega_{g}\in \left[d_{\min},d_{\max}\right]$. (iii) *Measurements and d–q frame*: the measured stator currents are expressed in the *d*–*q* frame through the Park transformation, which uses the electrical angle, assumed available. The rotational speed is not used by the observer, which provides an independent estimate of it from the current measurements only; this provides analytical redundancy for the speed. This is why the speed, used as the premise variable, is considered unmeasurable. (iv) *Faults*: actuator faults are additive voltage deviations on the *q*-axis input (inverter faults), and sensor faults are additive deviations on the *d*-axis current measurement; in both cases, they are time-varying signals (biases, drifts, transients or their successions), whose distribution matrices are given in Section 3.

The nonlinear dynamic model of the PMSG-based WECS is written as:

(7) $$\left\{\begin{array}{cc} \dot{x}\left(t\right) & =A(\omega_{g}\left(t\right))x\left(t\right)+Bu\left(t\right)\\ y(t) & =Cx(t) \end{array}\right.$$

where $x\left(t\right)={\left[\begin{array}{ccc} \omega_{g}\left(t\right) & i_{q}\left(t\right) & i_{d}\left(t\right) \end{array}\right]}^{T}$ is the state vector, $u\left(t\right)={\left[\begin{array}{cc} V_{q}\left(t\right) & V_{d}\left(t\right) \end{array}\right]}^{T}$ is the input vector, and the matrices are:

(8) $$A\left(\omega_{g}\left(t\right)\right)=\left[\begin{array}{ccc} \frac{-\Lambda -T_{0}\omega_{g}\left(t\right)}{J} & \frac{1.5\rho_{n}\phi_{mag}}{J} & 0\\ -\frac{\rho_{n}\phi_{mag}}{L} & -\frac{R_{s}}{L} & -\rho_{n}\omega_{g}\left(t\right)\\ 0 & \rho_{n}\omega_{g}\left(t\right) & -\frac{R_{s}}{L} \end{array}\right],\;B=\left[\begin{array}{cc} 0 & 0\\ \frac{1}{L} & 0\\ 0 & \frac{1}{L} \end{array}\right],\;C=\left[\begin{array}{ccc} 0 & 1 & 0\\ 0 & 0 & 1 \end{array}\right]$$

Only the stator currents $i_{q}$ and $i_{d}$ are measured by current sensors; the rotational speed $\omega_{g}$, used as the premise variable, is not measured. Consequently, the premise variable is unmeasurable, which motivates the use of estimated weights in the observer and the treatment of the resulting mismatch in Section 3.

### 2.3. Takagi–Sugeno Modeling of the PMSG-Based WECS

The nonlinear system (7) is converted into a T–S fuzzy model using the premise variable $x_{1}\left(t\right)=\omega_{g}\left(t\right)\in \left[d_{\min},d_{\max}\right]$ (sector nonlinearity approach [22]):

(9) $$\left\{\begin{array}{cc} \dot{x}\left(t\right) & =\sum_{i=1}^{2}\mu_{i}\left(\omega_{g}\left(t\right)\right)\left(A_{i}x\left(t\right)+B_{i}u\left(t\right)\right)\\ y(t) & =Cx(t) \end{array}\right.$$

with the membership functions

(10) $$\mu_{1}\left(\omega_{g}\right)={\mathrm{sat}}_{\left[0,1\right]}\;\left(\frac{d_{\max}-\omega_{g}}{d_{\max}-d_{\min}}\right),\;\mu_{2}\left(\omega_{g}\right)=1-\mu_{1}\left(\omega_{g}\right),$$

which satisfy $0\leq \mu_{i}\leq 1$ and $\mu_{1}+\mu_{2}=1$, and

(11) $$A_{1}=\left[\begin{array}{ccc} \frac{-\Lambda -T_{0}d_{\min}}{J} & \frac{1.5\rho_{n}\phi_{mag}}{J} & 0\\ -\frac{\rho_{n}\phi_{mag}}{L} & -\frac{R_{s}}{L} & -\rho_{n}d_{\min}\\ 0 & \rho_{n}d_{\min} & -\frac{R_{s}}{L} \end{array}\right],\;A_{2}=\left[\begin{array}{ccc} \frac{-\Lambda -T_{0}d_{\max}}{J} & \frac{1.5\rho_{n}\phi_{mag}}{J} & 0\\ -\frac{\rho_{n}\phi_{mag}}{L} & -\frac{R_{s}}{L} & -\rho_{n}d_{\max}\\ 0 & \rho_{n}d_{\max} & -\frac{R_{s}}{L} \end{array}\right]$$

$$B_{1}=B_{2}=B.$$

## 3. PI-TS Observer Formulation

### 3.1. Faulty System Model

The T–S fuzzy model of the PMSG-based wind turbine including sensor and actuator faults is written as:

(12) $$\left\{\begin{array}{cc} \dot{x}\left(t\right) & =\sum_{i=1}^{r}\mu_{i}\left(\omega_{g}\left(t\right)\right)\left(A_{i}x\left(t\right)+B_{i}u\left(t\right)+F_{a}f_{a}\left(t\right)\right)\\ y(t) & =Cx\left(t\right)+F_{s}f_{s}\left(t\right)+Hw\left(t\right) \end{array}\right.$$

where $f_{a}\left(t\right)$ and $f_{s}\left(t\right)$ are the actuator and sensor faults, respectively, and w(t) is the disturbance input.

The fault and disturbance distribution matrices are specified as follows. The actuator fault $f_{a}\left(t\right)\in R$ is an additive voltage fault on the *q*-axis input, $F_{a}={\left[\;0\;\;1/L\;\;0\;\right]}^{T}$, representing an inverter voltage fault. The sensor fault $f_{s}\left(t\right)\in R$ affects the *d*-axis current measurement, $F_{s}={\left[\;0\;\;1\;\right]}^{T}$, representing a measurement deviation of the current sensor (bias, drift or transient). The measurement disturbance w(t)∈R is distributed by $H={\left[\;0\;\;1\;\right]}^{T}$.

To estimate the actuator and sensor faults simultaneously, the output-filtering augmentation of [28,32] is used: a new state $\xi \left(t\right)\in R^{n_{y}}$, a filtered version of y(t), is defined by $\dot{\xi }\left(t\right)=-E\left(\xi \left(t\right)-y\left(t\right)\right)$, where −E is a stable matrix.

In the original system (12), the sensor fault $f_{s}\left(t\right)$ enters the output equation algebraically, so it cannot be handled by a classical unknown input observer, which only handles signals acting on the state dynamics. Injecting the measured output into the low-pass filter gives

(13) $$\dot{\xi }\left(t\right)=-E\;\xi \left(t\right)+ECx\left(t\right)+EF_{s}f_{s}\left(t\right)+EHw\left(t\right),$$

which shows that the sensor fault is transferred into the *dynamics* of the auxiliary state ξ(t). In the augmented state $X\left(t\right)={\left[\;x^{T}\left(t\right)\;\;\xi^{T}\left(t\right)\;\right]}^{T}$, the sensor fault therefore acts as an equivalent actuator-type fault through the input matrix $EF_{s}$, which makes its joint reconstruction with $f_{a}\left(t\right)$ possible within a single PI observer. An additional advantage is that the new output $Y\left(t\right)=\bar{C}X\left(t\right)=\xi \left(t\right)$ is no longer directly corrupted by $f_{s}\left(t\right)$. The filtering matrix *E* has a direct physical meaning: it sets the bandwidth of the output filter, i.e., how fast ξ(t) tracks y(t), and hence how fast the sensor fault is transferred into the augmented dynamics. With the selected value $E=10\;I_{2}$ (Section 4), the auxiliary state ξ tracks the measured currents with a time constant of 0.1 s, much faster than the turbine dynamics. A larger *E* transfers the sensor fault faster to the augmented dynamics, but enlarges the blocks EC, $EF_{s}$ and EH, and hence the observer gains; a smaller *E* slows down the reconstruction of the sensor fault. The selected value achieves a trade-off between these two effects.

The augmented system is written as:

(14) $$\left\{\begin{array}{c} \dot{X}\left(t\right)=\sum_{i=1}^{r}\mu_{i}\left(\omega_{g}\left(t\right)\right)\left({\bar{A}}_{i}X\left(t\right)+{\bar{B}}_{i}u\left(t\right)+{\bar{F}}_{i}f\left(t\right)\right)+\bar{H}w\left(t\right)\\ Y\left(t\right)=\bar{C}X\left(t\right) \end{array}\right.$$

where

(15) $$X\left(t\right)=\left[\begin{array}{c} x(t)\\ \xi (t) \end{array}\right]\in R^{\tilde{n}},\;f\left(t\right)=\left[\begin{array}{c} f_{a}\left(t\right)\\ f_{s}\left(t\right) \end{array}\right]\in R^{n_{f}}$$

with $\tilde{n}=n+n_{y}$ and $n_{f}=n_{a}+n_{s}$. The physical system is weighted by the true premise variable $\omega_{g}\left(t\right)$, whereas the observer defined below can only use its estimate ${\hat{\omega }}_{g}\left(t\right)$; this mismatch is the central difficulty addressed in Section 3.4. The extended matrices are:

(16) $${\bar{A}}_{i}=\left[\begin{array}{cc} A_{i} & 0\\ EC & -E \end{array}\right],\;{\bar{B}}_{i}=\left[\begin{array}{c} B_{i}\\ 0 \end{array}\right],\;{\bar{F}}_{i}=\left[\begin{array}{cc} F_{a} & 0\\ 0 & EF_{s} \end{array}\right],\;\bar{H}=\left[\begin{array}{c} 0\\ EH \end{array}\right],\;\bar{C}=\left[\begin{array}{cc} 0 & I_{n_{y}} \end{array}\right]$$

**Assumption 1.** 
*The fault signal $f\left(t\right)\in R^{n_{f}}$ is time-varying and has bounded successive derivatives up to order k; its kth derivative is bounded by $f_{0}$:*

(17) $$\left\{\begin{array}{c} f\left(t\right)=f_{1}\left(t\right),\;{\dot{f}}_{1}\left(t\right)=f_{2}\left(t\right),\;\ldots ,\;{\dot{f}}_{k-1}\left(t\right)=f_{k}\left(t\right)\\ ∥f_{k}\left(t\right)∥\leq f_{0} \end{array}\right.$$

*The disturbance w(t) is assumed to be bounded and of finite energy over the considered horizon, $w\in L_{2}\left[0,T\right]$*.

### 3.2. Structural Conditions

The joint reconstruction of states and faults requires the following two conditions, formulated on the augmented matrices introduced in Section 3.1 and Section 3.3.

**Assumption 2** 
(Detectability)**.** *For i=1,2, the pair $\left(A_{i},C\right)$ is detectable*.

**Assumption 3** 
(Fault reconstructability)**.** *For i=1,2,*

(18) $$\mathrm{rank}\left[\begin{array}{cc} {\bar{A}}_{i} & {\bar{F}}_{i}\\ \bar{C} & 0 \end{array}\right]=\tilde{n}+n_{f}.$$

*These conditions are verified numerically in Section 4*.

### 3.3. Observer Design

The PI observer structure of [28,29] is adopted, without the discontinuous compensation term used in [28]. It provides the states and the actuator and sensor faults simultaneously in the presence of an unmeasurable premise variable:

(19) $$\left\{\begin{array}{c} \dot{\hat{X}}\left(t\right)=\sum_{i=1}^{r}\mu_{i}\left({\hat{\omega }}_{g}\right)\left({\bar{A}}_{i}\hat{X}\left(t\right)+{\bar{B}}_{i}u\left(t\right)+{\bar{F}}_{i}\hat{f}\left(t\right)+K_{P,i}\;\left(Y\left(t\right)-\hat{Y}\left(t\right)\right)\right)\\ \hat{Y}\left(t\right)=\bar{C}\hat{X}\left(t\right)\\ \dot{\hat{f}}\left(t\right)=\sum_{i=1}^{r}\mu_{i}\left({\hat{\omega }}_{g}\right)\;K_{I,i}\;\left(Y\left(t\right)-\hat{Y}\left(t\right)\right)+{\hat{f}}_{1}\left(t\right)\\ {\dot{\hat{f}}}_{j}\left(t\right)=\sum_{i=1}^{r}\mu_{i}\left({\hat{\omega }}_{g}\right)\;K_{I,i}^{j}\;\left(Y\left(t\right)-\hat{Y}\left(t\right)\right)+{\hat{f}}_{j+1}\left(t\right),\;j=1,\ldots ,k-1 \end{array}\right.$$

where ${\hat{\omega }}_{g}={\hat{x}}_{1}$ is the estimated premise variable, $K_{P,i}\in R^{\tilde{n}\times n_{y}}$, $K_{I,i}\in R^{n_{f}\times n_{y}}$ and $K_{I,i}^{j}\in R^{n_{f}\times n_{y}}$ are rule-dependent proportional and integral gains to be designed.

Since the premise variable $\omega_{g}$ is not measured, the observer evaluates the membership functions at the estimated speed, and the mismatch ${\bar{\mu }}_{i}=\mu_{i}\left(\omega_{g}\right)-\mu_{i}\left({\hat{\omega }}_{g}\right)$ enters the estimation error dynamics. This mismatch can be compensated by a discontinuous term whose gain is set by worst-case bounds of the mismatch [28]; for the present model, a gain consistent with these bounds would lead to a very high-gain switching injection. The mismatch is therefore treated here as a bounded input acting on the error dynamics, and its effect is attenuated through the LMI conditions of Theorem 1, following the $L_{2}$ approach of [30].

Based on Assumption 1, the augmented forms of the T–S model and of the PI observer are:

(20) $$\left\{\begin{array}{c} \dot{\bar{X}}\left(t\right)=\sum_{i=1}^{r}\mu_{i}\left(\omega_{g}\right)\left(A_{i}\bar{X}\left(t\right)+B_{i}u\left(t\right)\right)+Gw\left(t\right)+Rf_{k}\left(t\right)\\ Y\left(t\right)=C\bar{X}\left(t\right)\\ \dot{\hat{\bar{X}}}\left(t\right)=\sum_{i=1}^{r}\mu_{i}\left({\hat{\omega }}_{g}\right)\left(A_{i}\hat{\bar{X}}\left(t\right)+B_{i}u\left(t\right)+K_{i}\left(Y\left(t\right)-\hat{Y}\left(t\right)\right)\right)\\ \hat{Y}\left(t\right)=C\hat{\bar{X}}\left(t\right) \end{array}\right.$$

where

(21) $$\bar{X}\left(t\right)=\left[\begin{array}{c} X(t)\\ f(t)\\ f_{1}\left(t\right)\\ \vdots \\ f_{k-1}\left(t\right) \end{array}\right],\;\hat{\bar{X}}\left(t\right)=\left[\begin{array}{c} \hat{X}\left(t\right)\\ \hat{f}\left(t\right)\\ {\hat{f}}_{1}\left(t\right)\\ \vdots \\ {\hat{f}}_{k-1}\left(t\right) \end{array}\right]$$

with $\bar{X}\left(t\right)\in R^{n_{k}}$, $n_{k}=\tilde{n}+k\;n_{f}$, and 

(22) $$A_{i}=\left[\begin{array}{ccccc} {\bar{A}}_{i} & {\bar{F}}_{i} & 0 & \ldots & 0\\ 0 & 0 & I_{n_{f}} & \ldots & 0\\ 0 & 0 & 0 & \ddots & 0\\ \vdots & \vdots & \vdots & \; & I_{n_{f}}\\ 0 & 0 & 0 & \ldots & 0 \end{array}\right],\;B_{i}=\left[\begin{array}{c} {\bar{B}}_{i}\\ 0\\ \vdots \\ 0 \end{array}\right],\;K_{i}=\left[\begin{array}{c} K_{P,i}\\ K_{I,i}\\ K_{I,i}^{1}\\ \vdots \\ K_{I,i}^{k-1} \end{array}\right],\;G=\left[\begin{array}{c} \bar{H}\\ 0\\ \vdots \\ 0 \end{array}\right]\in R^{n_{k}\times n_{w}}$$

(23) $$R={\left[\begin{array}{cccc} 0 & 0 & \ldots & I_{n_{f}} \end{array}\right]}^{T}\in R^{n_{k}\times n_{f}},\;C=\left[\begin{array}{cccc} \bar{C} & 0 & \ldots & 0 \end{array}\right]\in R^{n_{y}\times n_{k}}$$

Defining the augmented estimation error  $\tilde{e}=\bar{X}-\hat{\bar{X}}$ and noting that $Y-\hat{Y}=C\tilde{e}$, its dynamics are

(24) $$\dot{\tilde{e}}=\sum_{i=1}^{r}\mu_{i}\left(\omega_{g}\right)\left(A_{i}\bar{X}+B_{i}u\right)-\sum_{i=1}^{r}\mu_{i}\left({\hat{\omega }}_{g}\right)\left(A_{i}\hat{\bar{X}}+B_{i}u+K_{i}C\tilde{e}\right)+Rf_{k}+Gw$$

Adding and subtracting $\sum_{i}\mu_{i}\left({\hat{\omega }}_{g}\right)\left(A_{i}\bar{X}+B_{i}u\right)$, we obtain

(25) $$\sum_{i=1}^{r}\mu_{i}\left(\omega_{g}\right)\left(A_{i}\bar{X}+B_{i}u\right)=\sum_{i=1}^{r}\mu_{i}\left({\hat{\omega }}_{g}\right)\left(A_{i}\bar{X}+B_{i}u\right)+\Delta A\;\bar{X}+\Delta B\;u$$

with $\Delta A=\sum_{i}{\bar{\mu }}_{i}A_{i}$, $\Delta B=\sum_{i}{\bar{\mu }}_{i}B_{i}$ and ${\bar{\mu }}_{i}=\mu_{i}\left(\omega_{g}\right)-\mu_{i}\left({\hat{\omega }}_{g}\right)$. The input terms cancel and $A_{i}\bar{X}-A_{i}\hat{\bar{X}}=A_{i}\tilde{e}$, so that

(26) $$\dot{\tilde{e}}\left(t\right)=\sum_{i=1}^{r}\mu_{i}\left({\hat{\omega }}_{g}\right)\;{\bar{A}}_{i}\;\tilde{e}\left(t\right)+WE\left(t\right)+d(t)$$

where

(27) $${\bar{A}}_{i}=A_{i}-K_{i}C,\;W=\left[\begin{array}{cc} R & G \end{array}\right],\;E\left(t\right)=\left[\begin{array}{c} f_{k}\left(t\right)\\ w(t) \end{array}\right],\;d\left(t\right)=\Delta A\;\bar{X}\left(t\right)+\Delta B\;u\left(t\right)$$

The mismatch multiplies the true augmented state $\bar{X}$ and the input *u*, and not the estimation error $\tilde{e}$: it acts on the error dynamics as the input d(t), which is bounded whenever $\bar{X}$ and *u* are bounded on the operating domain.

**Remark 1.** 
*Since $\mu_{i}\left(\omega_{g}\right)$ and $\mu_{i}\left({\hat{\omega }}_{g}\right)$ both satisfy the convex sum property, $\sum_{i=1}^{r}{\bar{\mu }}_{i}=0$ and $-1\leq {\bar{\mu }}_{i}\leq 1$; the ${\bar{\mu }}_{i}$ are therefore not convex weights, and ΔA, ΔB cannot be bounded directly by the norms of the local matrices. For an r-rule model,*

(28) $$\Delta A=\sum_{i=1}^{r-1}{\bar{\mu }}_{i}\;\left(A_{i}-A_{r}\right),\;\Delta B=\sum_{i=1}^{r-1}{\bar{\mu }}_{i}\;\left(B_{i}-B_{r}\right),$$

*so that, with $|{\bar{\mu }}_{i}|\leq 1$ and the triangle inequality, the mismatch matrices are bounded by the norms of the differences between local matrices:*

(29) $$∥\Delta A∥\leq \delta_{1}:=\sum_{i=1}^{r-1}∥A_{i}-A_{r}{∥}_{2},\;∥\Delta B∥\leq \delta_{2}:=\sum_{i=1}^{r-1}{∥B_{i}-B_{r}∥}_{2}.$$

*For the two-rule model considered here*, ${\bar{\mu }}_{2}=-{\bar{\mu }}_{1}$ *and* (28) *reduces to*

(30) $$\Delta A={\bar{\mu }}_{1}\;N,\;N:=A_{1}-A_{2},\;\Delta B={\bar{\mu }}_{1}\left(B_{1}-B_{2}\right)=0,$$

*the last identity following from* $B_{1}=B_{2}$. *The input mismatch term therefore vanishes identically, and the bounds* (29) *become* $\delta_{1}={∥A_{1}-A_{2}∥}_{2}$ *and* $\delta_{2}=0$. *Moreover, since* $\mu_{1}$ *in* (10) *is Lipschitz with constant* $1/(d_{\max}-d_{\min})$, *the mismatch satisfies*

(31) $$|{\bar{\mu }}_{1}|\leq \frac{|\omega_{g}-{\hat{\omega }}_{g}|}{d_{\max}-d_{\min}},$$

*so that it vanishes as the speed estimate converges. Consequently, if the true augmented state and the input remain bounded on the operating domain,* $∥\bar{X}\left(t\right)∥\leq \rho_{X}$ *and* $∥u\left(t\right)∥\leq \rho_{u}$, *the mismatch input* (27) *satisfies*

(32) $$∥d\left(t\right)∥\leq ∥\Delta A∥\;∥\bar{X}\left(t\right)∥+∥\Delta B∥\;∥u\left(t\right)∥\leq \bar{d}:=\delta_{1}\rho_{X}+\delta_{2}\rho_{u}.$$

*For a given model, a smaller value of $\bar{d}$ can be obtained by exploiting the structure of N (see Section 4)*.

**Lemma 1** 
(Young’s Inequality)**.** *For any matrices X and Y of appropriate dimensions and any scalar α>0 [22]:*

(33) $$X^{T}Y+Y^{T}X\leq \alpha \;X^{T}X+\alpha^{-1}\;Y^{T}Y$$

### 3.4. Convergence and $L_{2}$ Conditions

The observer gains $K_{i}$ are computed from LMI conditions in which the mismatch input d(t) is treated as a bounded input, following the $L_{2}$ approach of [30], and in which a decay-rate constraint is included. Introducing a positive scalar β into the Lyapunov inequality ensures an exponential convergence rate of at least β in the absence of inputs [33]; this appears as the term 2βP in $\varphi_{i}$ below.

**Theorem 1.** 
*Consider the error dynamics* (26), *and assume that* $∥E\left(t\right)∥\leq \bar{E}$ *and* $∥d\left(t\right)∥\leq \bar{d}$ *for all* t≥0*, where* $\bar{d}$ *is given, for instance, by* (32). *For a prescribed* β>0*, if there exist a matrix* $P=P^{T}>0$*, matrices* $M_{i}$ *and positive scalars λ and μ such that, for all* i=1,…,r,

(34) $$\left(\begin{array}{ccccc} \varphi_{i} & PR & PG & P & I_{n_{k}}\\ {}* & -\mu I_{n_{f}} & 0 & 0 & 0\\ {}* & * & -\mu I_{n_{w}} & 0 & 0\\ {}* & * & * & -\lambda I_{n_{k}} & 0\\ {}* & * & * & * & -\mu I_{n_{k}} \end{array}\right)<0,\;\varphi_{i}=H\left(PA_{i}-M_{i}C\right)+2\beta P,$$

*where* $H\left(X\right)=X+X^{T}$*, then, with the gains* $K_{i}=P^{-1}M_{i}$ *and* $V={\tilde{e}}^{T}P\tilde{e}$:

*(i)*  $\dot{V}+2\beta V+\mu^{-1}∥\tilde{e}{∥}^{2}-\mu ∥E{∥}^{2}-\lambda {∥d∥}^{2}\leq 0$ *along the trajectories of* (26); *in particular, if* E=0 *and* d=0, $∥\tilde{e}\left(t\right)∥\leq \sqrt{\lambda_{\max}\left(P\right)/\lambda_{\min}\left(P\right)}\;e^{-\beta t}∥\tilde{e}\left(0\right)∥$;
*(ii)*  *the estimation error is uniformly ultimately bounded:* ${\mathrm{lim sup}}_{t\to \infty }∥\tilde{e}\left(t\right)∥\leq b:=\sqrt{\left(\lambda {\bar{d}}^{2}+\mu {\bar{E}}^{2}\right)/\left(2\beta \lambda_{\min}\left(P\right)\right)}$*, and, for any* $b^{\prime }>b$*, the error enters the ball of radius* $b^{\prime }$ *in finite time and remains in it*.
*(iii)*  *for any* T>0, $∥\tilde{e}{∥}_{L_{2}\left[0,T\right]}^{2}\leq \mu^{2}{∥E∥}_{L_{2}\left[0,T\right]}^{2}+\mu \lambda {∥d∥}_{L_{2}\left[0,T\right]}^{2}+\mu V\left(0\right)$.

*The smallest attenuation level* $\mu^{*}$ *is obtained by minimizing μ subject to* (34).

**Proof.**  Consider the Lyapunov function $V={\tilde{e}}^{T}P\tilde{e}$, with $P=P^{T}>0$. Since *P* is symmetric, $\dot{V}={\dot{\tilde{e}}}^{T}P\tilde{e}+{\tilde{e}}^{T}P\dot{\tilde{e}}=2{\tilde{e}}^{T}P\dot{\tilde{e}}$. Substituting (26) and using $2x^{T}Mx=x^{T}H\left(M\right)x$,

(35) $$\dot{V}=\sum_{i=1}^{r}\mu_{i}\left({\hat{\omega }}_{g}\right)\;{\tilde{e}}^{T}H\left(P{\bar{A}}_{i}\right)\tilde{e}+2{\tilde{e}}^{T}PWE+2{\tilde{e}}^{T}Pd.$$

Applying Lemma 1 to the mismatch term, with $X=P\tilde{e}$, Y=d and the scalar $\lambda^{-1}$, gives

(36) $$2{\tilde{e}}^{T}Pd\leq \lambda^{-1}{\tilde{e}}^{T}P^{2}\tilde{e}+\lambda \;d^{T}d.$$

This inequality holds for any λ>0; λ is therefore a decision variable of the LMI, and no relation with another scalar has to be satisfied.

Let $J=\dot{V}+2\beta V+\mu^{-1}{\tilde{e}}^{T}\tilde{e}-\mu \;E^{T}E-\lambda \;d^{T}d$. Substituting (35) and (36), the terms $\lambda d^{T}d$ cancel. Since $\sum_{i}\mu_{i}\left({\hat{\omega }}_{g}\right)=1$, the terms that do not depend on *i* can be written inside the sum, and, with $\zeta ={\left[\;{\tilde{e}}^{T}\;\;E^{T}\;\right]}^{T}$,

(37) $$J\leq \sum_{i=1}^{r}\mu_{i}\left({\hat{\omega }}_{g}\right)\;\zeta^{T}\Omega_{i}\zeta ,\;\Omega_{i}=\left[\begin{array}{cc} \Psi_{i} & PW\\ {}* & -\mu I \end{array}\right],\;\Psi_{i}=H\left(P{\bar{A}}_{i}\right)+2\beta P+\lambda^{-1}P^{2}+\mu^{-1}I_{n_{k}}.$$

Setting $M_{i}=PK_{i}$, one has $P{\bar{A}}_{i}=PA_{i}-M_{i}C$, so that $\Psi_{i}=\varphi_{i}+\lambda^{-1}P^{2}+\mu^{-1}I_{n_{k}}$. Since $\mathrm{diag}(-\lambda I_{n_{k}},-\mu I_{n_{k}})<0$, the Schur complement [33] shows that $\Omega_{i}<0$ is equivalent to (34). Hence, if (34) holds for all *i*, each $\zeta^{T}\Omega_{i}\zeta \leq 0$ and, the weights $\mu_{i}\left({\hat{\omega }}_{g}\right)$ being non-negative, J≤0, which is (i). If E=0 and d=0, (i) gives $\dot{V}\leq -2\beta V$, hence $V\left(t\right)\leq e^{-2\beta t}V\left(0\right)$, and $\lambda_{\min}\left(P\right)∥\tilde{e}{∥}^{2}\leq V\leq \lambda_{\max}\left(P\right){∥\tilde{e}∥}^{2}$ gives the exponential bound.

To prove (ii), it follows from (i), $∥E∥\leq \bar{E}$ and $∥d∥\leq \bar{d}$ that $\dot{V}\leq -2\beta V+c$ with $c=\lambda {\bar{d}}^{2}+\mu {\bar{E}}^{2}$. Then $\frac{d}{dt}\left(e^{2\beta t}\left(V-c/\left(2\beta \right)\right)\right)=e^{2\beta t}\left(\dot{V}+2\beta V-c\right)\leq 0$, so that (comparison lemma, Lemma 3.4 of [34])

(38) $$V\left(t\right)\leq e^{-2\beta t}V\left(0\right)+\frac{c}{2\beta }\left(1-e^{-2\beta t}\right),\;{∥\tilde{e}\left(t\right)∥}^{2}\leq \frac{e^{-2\beta t}V\left(0\right)}{\lambda_{\min}\left(P\right)}+b^{2}.$$

Hence ${\mathrm{lim sup}}_{t\to \infty }∥\tilde{e}\left(t\right)∥\leq b$. For any $b^{\prime }>b$, $∥\tilde{e}\left(t\right)∥\leq b^{\prime }$ for all $t\geq t^{*}$, where $t^{*}$ is any time such that $e^{-2\beta t^{*}}V\left(0\right)\leq \lambda_{\min}\left(P\right)\left(b^{\prime 2}-b^{2}\right)$. This proves (ii).

Finally, since 2βV≥0, (i) gives $\dot{V}+\mu^{-1}∥\tilde{e}{∥}^{2}\leq {\mu ∥E∥}^{2}+\lambda {∥d∥}^{2}$. Integrating over [0,T], using V(T)≥0 and multiplying by μ>0 gives (iii).    □

**Remark 2.** 
*In* $\bar{d}$, $|{\bar{\mu }}_{1}|\leq 1$ *is used, whereas, by* (31), $∥d\left(t\right)∥\leq ∥N\bar{X}\left(t\right)∥\;|\omega_{g}-{\hat{\omega }}_{g}|/\left(d_{\max}-d_{\min}\right)$ *for the two-rule model, so that the mismatch input vanishes as the speed estimate converges. The bounds (ii) and (iii) hold for every trajectory of the operating domain, and μ is the attenuation level of* E *in the absence of mismatch. When the premise variable is measured,* d=0 *: (i) then gives the exponential convergence of the error when* E=0*, and (iii) reduces to the nominal induced-*$L_{2}$ *attenuation* $∥\tilde{e}{∥}_{L_{2}\left[0,T\right]}^{2}\leq \mu^{2}{∥E∥}_{L_{2}\left[0,T\right]}^{2}+\mu V\left(0\right)$.

## 4. Simulation Results and Discussion

To evaluate the proposed PI-TS observer, numerical simulations were carried out in MATLAB R2025b (MathWorks, Natick, MA, USA) under the modeling assumptions of Section 2, with the physical parameters of Table 1. The conditions of Theorem 1 were solved with YALMIP and the SDPT3 solver (default tolerances): the gains of Appendix A satisfy them for i=1,2; the minimum of μ with $\lambda =3\times 10^{4}$ is $\mu^{*}=1.875$, and the matrix *P*, whose extreme eigenvalues are given in Table 2, satisfies them strictly for μ=1.9, the largest eigenvalue of the LMI matrices being $-1.2\times 10^{-3}$. For the mismatch bound, the structure of N in (30) is exploited: N only acts on the state *x*, with $N\bar{X}=\left(d_{\max}-d_{\min}\right)\;{\left[\;T_{0}\omega_{g}/J,\;\rho_{n}i_{d},\;-\rho_{n}i_{q},\;0,\ldots ,0\;\right]}^{T}$. Hence, if $|\omega_{g}|\leq \rho_{\omega }$ and $∥{\left[\;i_{q}\;\;i_{d}\;\right]}^{T}∥\leq \rho_{i}$, $∥d\left(t\right)∥\leq |{\bar{\mu }}_{1}|\;∥N\bar{X}∥\leq \bar{d}=\left(d_{\max}-d_{\min}\right)\sqrt{{\left(T_{0}\rho_{\omega }/J\right)}^{2}+\rho_{n}^{2}\rho_{i}^{2}}$, a value smaller than the general bound $\delta_{1}\rho_{X}$ of (32). With the values of Table 2, $\bar{d}=886$ and $b=3.6\times 10^{6}$ for E=0. Along the simulated trajectory of Scenario 1, ∥d(t)∥ is at most 80 during the initial transient and at most 5.3 for t≥2 s (Remark 2). The continuous-time system and observer are integrated with ode15s. The physical parameters of the model are given in Table 1, all design and simulation parameters in Table 2, and the obtained gains in Appendix A. The T–S fuzzy model is defined over $\omega_{g}\left(t\right)\in \left[d_{\min},d_{\max}\right]=\left[0,10\right]$ rad/s, a range that covers the entire simulated trajectory. The generator operates in open loop. The input voltages are constant, $V_{q}=V_{d}=10$ V, the simulation horizon is T=100 s, and the measurement disturbance is w(t)=0.1sin(2t)+0.05sin(15t)+0.03sin(50t) A. The system starts at rest, x(0)=0, and the observer is deliberately initialized at $\hat{x}\left(0\right)={\left[1,\;1,\;1\right]}^{T}$ in order to evaluate the convergence of the estimation error. A measurement disturbance with a peak amplitude of 0.18 A (about 2% of the steady-state *d*-axis current) acts on the $i_{d}$ channel over the entire horizon (Figure 1); this deterministic multi-frequency disturbance emulates measurement variations at several time scales.

In the three scenarios, the faults are test profiles built as sums of transitions. A transition moves the fault from one level to another, by an amount *M*, not instantaneously as a step, but over a finite duration $T_{r}$ starting at time $t_{0}$; an instantaneous step would have an unbounded second derivative and would not satisfy Assumption 1. Each transition is generated with a bounded second derivative, $\ddot{f}\left(s\right)=\left(2\pi M/T_{r}^{2}\right)\sin\left(2\pi s/T_{r}\right)$, $s=t-t_{0}\in \left[0,T_{r}\right]$, which gives $f\left(s\right)=M\;[\;s/T_{r}-\sin\left(2\pi s/T_{r}\right)/\left(2\pi \right)\;]$ and $∥\ddot{f}∥\leq 2\pi |M|/T_{r}^{2}$, so that Assumption 1 is satisfied.

The results are obtained in simulation and illustrate the behavior of the observer on the nonlinear PMSG model. They are organized into three scenarios, which use the same gains, the same disturbance and the same initialization. Scenario 1 considers simultaneous actuator and sensor faults: a deviation of the inverter voltage on the $V_{q}$ input and a deviation of the $i_{d}$ current measurement, each evolving through successive levels of different amplitudes and durations before returning to zero. Scenario 2 considers actuator faults only, and Scenario 3 sensor faults only, in three forms: a bias, a drift and a transient, with three occurrences per form.

The structural conditions of Section 3.2 are satisfied at both vertices: $\left(A_{i},C\right)$ is observable, and the rank in condition (18) equals $\tilde{n}+n_{f}=7$.

### 4.1. Scenario 1: Estimation of Simultaneous Actuator and Sensor Faults

This scenario evaluates the reconstruction of the states and of two simultaneous faults under an unmeasurable premise variable. The actuator fault $f_{a}\left(t\right)$, with a maximum amplitude of −4.5 V, is injected on the $V_{q}$ channel over [25,68] s; the sensor fault $f_{s}\left(t\right)$, with a maximum amplitude of 2.5 A, affects the $i_{d}$ measurement over [40,82] s. Both faults are therefore active simultaneously over [40,68] s. Each fault is a sum of five transitions of different amplitudes and durations (Table 3), which gives $f_{0}=0.982$.

Figure 2 shows the states $\omega_{g}$, $i_{q}$ and $i_{d}$ and their estimates. The unmeasured rotational speed is reconstructed from the current measurements only: the estimate converges from the erroneous initialization and then tracks the true state through the fault windows.

Figure 3 presents the fault reconstruction. The estimated actuator fault tracks the injected profile over [25,68] s, and the estimated sensor fault reproduces the injected profile over [40,82] s, including over the interval [40,68] s where both faults are active simultaneously.

Figure 4, Figure 5 and Figure 6 distinguish the output quantities. The curve $\hat{y}=C\hat{x}+F_{s}{\hat{f}}_{s}$ includes the estimated sensor fault and therefore reproduces the corrupted measurement *y* (Figure 4). On the *d*-axis, the measurement reaches about 11.35 A during the window [40,82] s, while the reconstructed output $C\hat{x}$ remains equal to the true fault-free current of about 8.85 A (Figure 5). The reconstruction error remains below $3\times 10^{-5}$ A on the *d*-axis and reacts only to the actuator-fault transitions (Figure 6a). The residual $r_{d}$ contains the ripple due to *w*, of the order of $10^{-4}$ A (Figure 6b). Since the sensor fault and the disturbance act on the same measurement channel, ${\hat{f}}_{s}$ reconstructs $f_{s}$ together with the part of *w* lying within the observer bandwidth, hence the ±0.17 A ripple observed in Figure 3. The state estimate, and therefore $C\hat{x}$, is not affected.

Figure 7 presents the state estimation errors $e_{x}=x-\hat{x}$. After the initial transient, they remain bounded during the fault injection windows [25,68] s and [40,82] s; their RMSEs are given in Table 4.

The quantitative indices are reported in Table 4 and Table 5. The RMSEs are computed as time-averaged integrals: after the initial transient (t≥2 s) for the states, over the injection window for the Scenario 1 faults, and over the occurrence windows for Scenarios 2 and 3; the maximum error is $\max|f-\hat{f}|$ over the same windows. The convergence time is the first instant after which the error remains within 5% of its initial value [35], and the reconstruction delay is defined with the same criterion. The empirical $L_{2}$ gain is defined as $\gamma_{\mathrm{emp}}=∥\tilde{e}{∥}_{L_{2}\left[2,T\right]}/{∥E∥}_{L_{2}\left[2,T\right]}$, with $E={\left[\;{\ddot{f}}^{T}\;\;w\;\right]}^{T}$ (k=2) and *T* the end of the simulation horizon; it is computed for the simulated disturbances and compared with the attenuation level $\mu^{*}$ of Theorem 1.

The unmeasured speed is reconstructed with an RMSE below 0.1% of its operating value and converges in 2.13 s from the erroneous initialization, while the output residuals remain of the order of $10^{-4}$ A (Figure 6b). The actuator fault is reconstructed with an RMSE of about 0.2% of its maximum amplitude and without measurable delay. For the sensor fault, the RMSE of $8.2\times 10^{-2}$ A is dominated by the ±0.17 A ripple due to the disturbance. The empirical $L_{2}$ gain (0.58) remains below the attenuation level $\mu^{*}=1.875$ of Theorem 1.

### 4.2. Scenario 2: Actuator Fault Estimation

This scenario evaluates the observer in the presence of actuator faults only, the measurement channel remaining healthy ($f_{s}\left(t\right)=0$). The three fault forms are injected with three occurrences each, with different amplitudes, signs and durations, each occurrence returning to zero; the bound $f_{0}$ of each profile is given in Table 5. The biases of −2, +2 and −4 V have plateaus over [13,21], [38,46] and [63,71] s (Figure 8a). The drifts are a decrease of −2 V over 15 s starting at t=10 s, an increase of +2 V over 20 s starting at t=40 s and a decrease of −3 V over 12 s starting at t=72 s, each returning to zero within 3 to 4 s (Figure 8b). The transients, with amplitudes of −3, +2 and −5 V, last a few seconds and are centered at t=23, 47.5 and 74 s (Figure 8c). In all three cases, the estimate tracks the injected profile and returns to zero between occurrences, with an RMSE of at most $3.2\times 10^{-2}$ V and a maximum error of at most $6.1\times 10^{-2}$ V (Table 5).

### 4.3. Scenario 3: Sensor Fault Estimation

This scenario evaluates the observer in the presence of sensor faults only, the actuator channel remaining healthy ($f_{a}\left(t\right)=0$). The same three fault forms are injected on the sensor channel, with three occurrences each. The biases of +0.5, −1.0 and +1.5 A have plateaus over [14,22], [39,47] and [66,74] s (Figure 9a). The drifts are an increase of +0.8 A over 15 s starting at t=10 s, a decrease of −1.2 A over 20 s starting at t=40 s and an increase of +1.5 A over 12 s starting at t=72 s, each returning to zero within 3 to 4 s (Figure 9b). The transients, with amplitudes of +1.0, −0.8 and +1.5 A, are centered at t=23, 47.5 and 74 s (Figure 9c). In all three cases, the estimate tracks the injected profile up to the ±0.17 A ripple due to the disturbance (Scenario 1): the RMSE ($8.2\times 10^{-2}$ A) and the maximum error (0.17 A) are the same for the three forms, since they are set by this ripple (Table 5).

## 5. Conclusions

This paper addressed the simultaneous estimation of actuator and sensor faults in a PMSG-based wind energy conversion system intended for isolated microgrids, in a configuration where only the stator currents are measured and the generator speed, the premise variable of the T–S fuzzy model, is not measured. The sensor fault is transferred into the dynamics of an augmented state by filtering the measured output, and an augmented fuzzy proportional–integral observer was adapted to this configuration to jointly reconstruct the states and both faults.

For this configuration, the structural conditions of detectability and fault reconstructability were stated and verified. The membership-function mismatch, which multiplies the true augmented state and is bounded through the differences between local matrices, was treated as a bounded input. This yields sufficient LMI conditions for the uniform ultimate boundedness of the estimation error, with a prescribed decay rate and an $L_{2}$ attenuation level.

The approach was evaluated in simulation on a small-scale PMSG model, under a persistent measurement disturbance, with simultaneous actuator and sensor faults and then biases, drifts and transients on each channel. The unmeasured speed is reconstructed with an RMSE of $4.7\times 10^{-3}$ rad/s, and the simultaneous faults with RMSEs of about 0.2% ($f_{a}$) and 3% ($f_{s}$) of their maximum amplitudes. In all scenarios, the empirical $L_{2}$ gain, at most 0.92, remained below the attenuation level of 1.875 obtained from the LMI conditions. These results illustrate, in simulation, the applicability of this framework to a physical energy conversion system.

The study relies on a simplified direct-drive PMSG model operating below rated wind speed under MPPT. The next steps are simulation within an isolated microgrid framework, in software-in-the-loop (SIL) and then hardware-in-the-loop (HIL) settings, and the integration of the estimator into an active fault-tolerant control scheme.

## Figures and Tables

**Figure 1:**
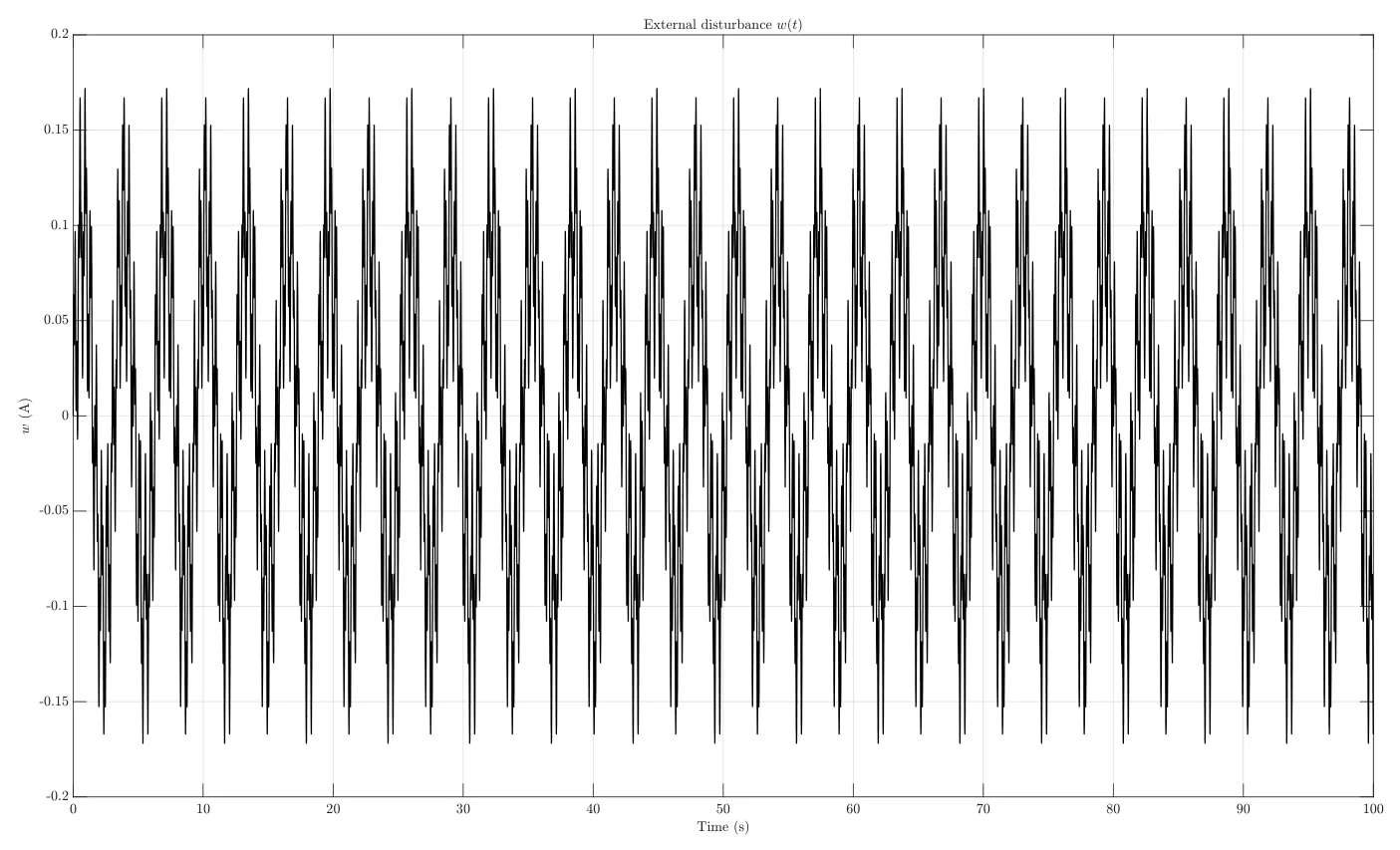
External measurement disturbance w(t) (peak amplitude of 0.18 A, components at 2, 15 and 50 rad/s) applied to the $i_{d}$ channel over the entire simulation horizon.

**Figure 2:**
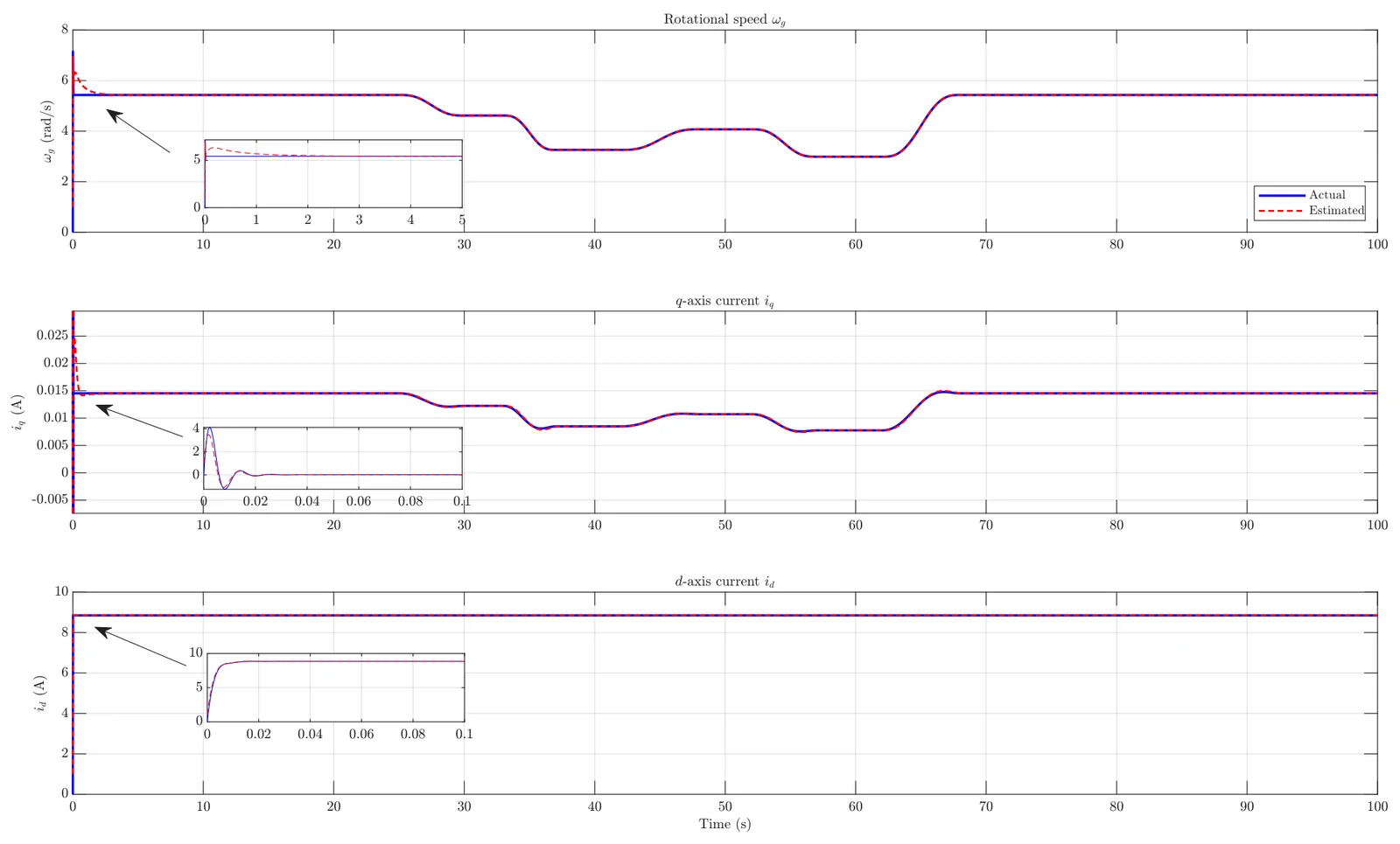
True states (solid blue line) and their estimates (dashed red line): rotational speed $\omega_{g}$ (**top**), *q*-axis current $i_{q}$ (**middle**) and *d*-axis current $i_{d}$ (**bottom**). The insets zoom in on the initial transient (5 s for $\omega_{g}$, 0.1 s for the currents).

**Figure 3:**
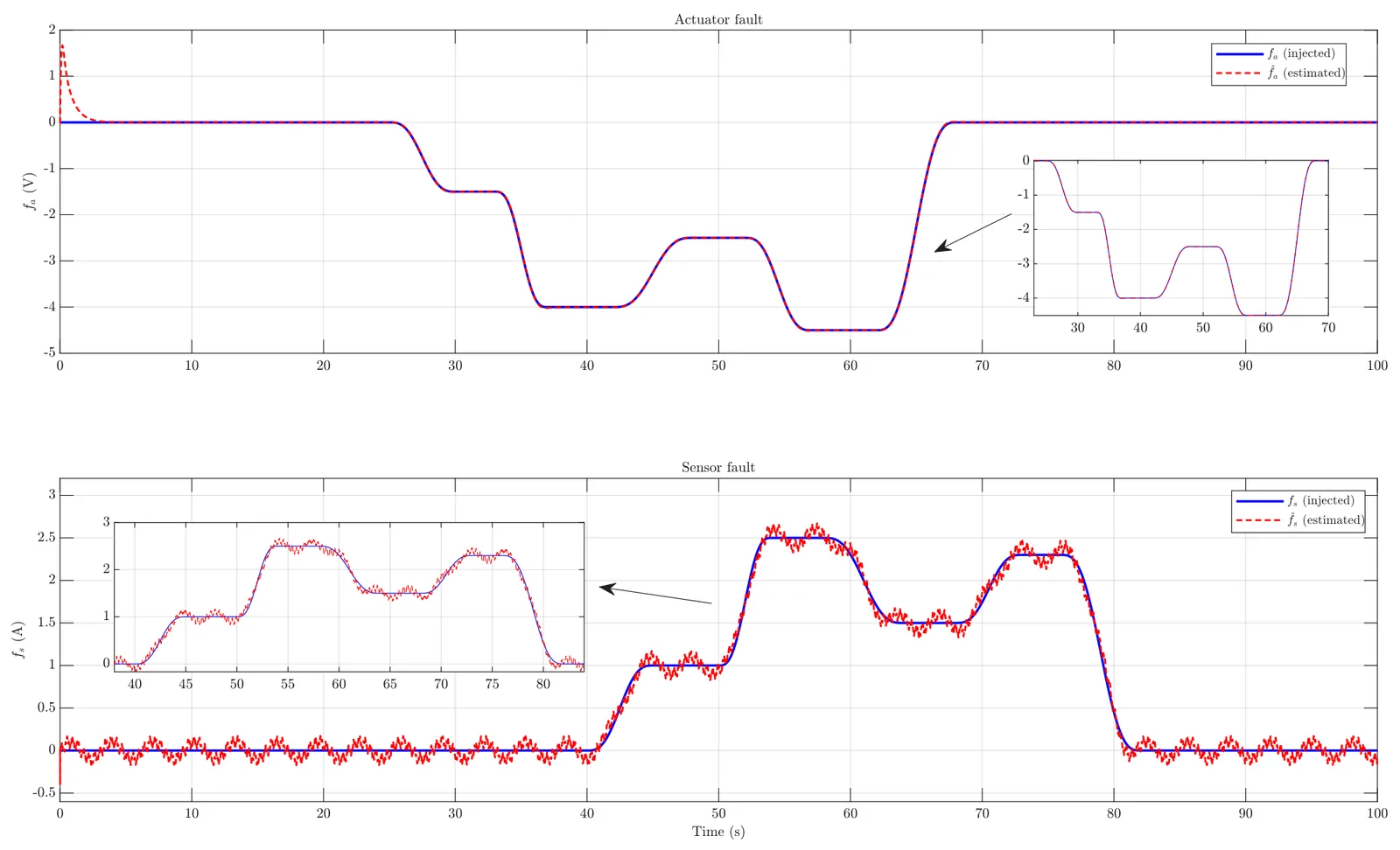
Reconstruction of the simultaneous faults: actuator fault with a maximum amplitude of −4.5 V over [25,68] s (**top**) and sensor fault with a maximum amplitude of 2.5 A over [40,82] s (**bottom**). Solid blue line: injected fault; dashed red line: estimated fault. The ripple on ${\hat{f}}_{s}$ is due to the disturbance, which acts on the same measurement channel; the peak of ${\hat{f}}_{a}$ at t≈0 is due to the erroneous initialization of the observer.

**Figure 4:**
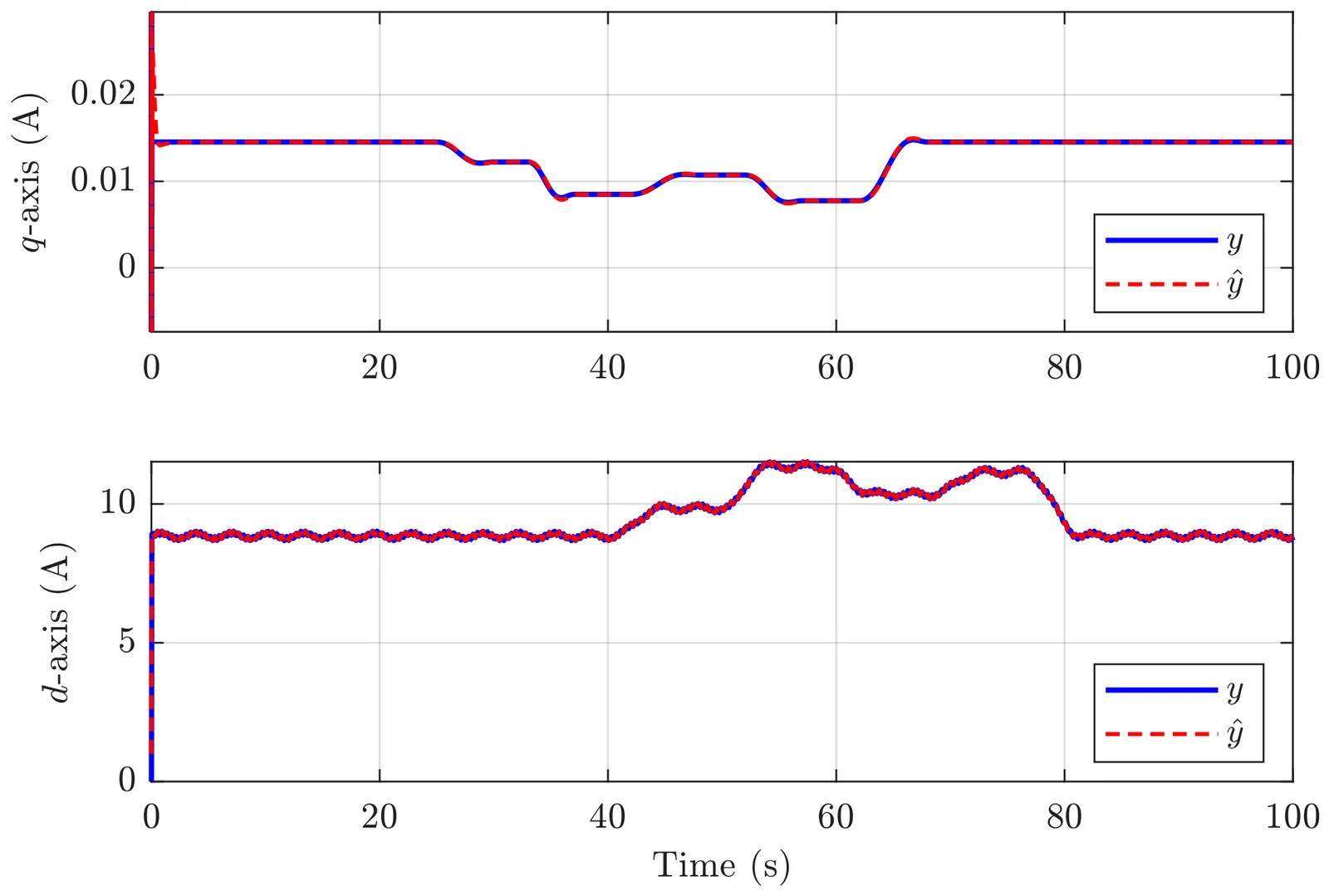
Corrupted measurement $y=Cx+F_{s}f_{s}+Hw$ and its estimate $\hat{y}=C\hat{x}+F_{s}{\hat{f}}_{s}$ ((**top**): *q*-axis; (**bottom**): *d*-axis). The sensor fault and the disturbance affect only the *d*-axis; the variations in the *q*-axis over [25,68] s are due to the actuator fault.

**Figure 5:**
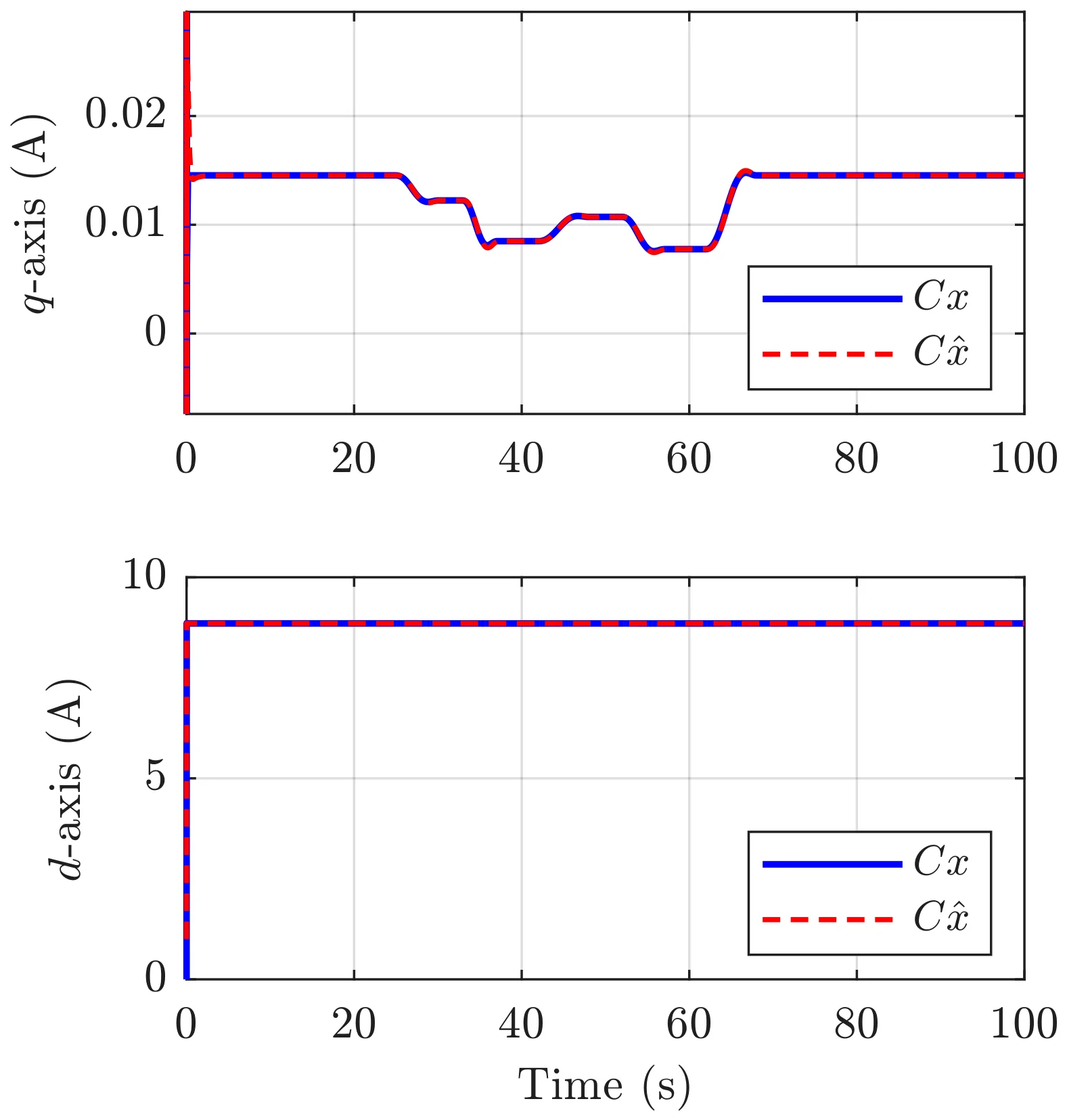
Fault-free output Cx and its reconstruction $C\hat{x}$ ((**top**): *q*-axis; (**bottom**): *d*-axis). On the *d*-axis, $C\hat{x}$ remains equal to the true current during the sensor fault window [40,82] s.

**Figure 6:**
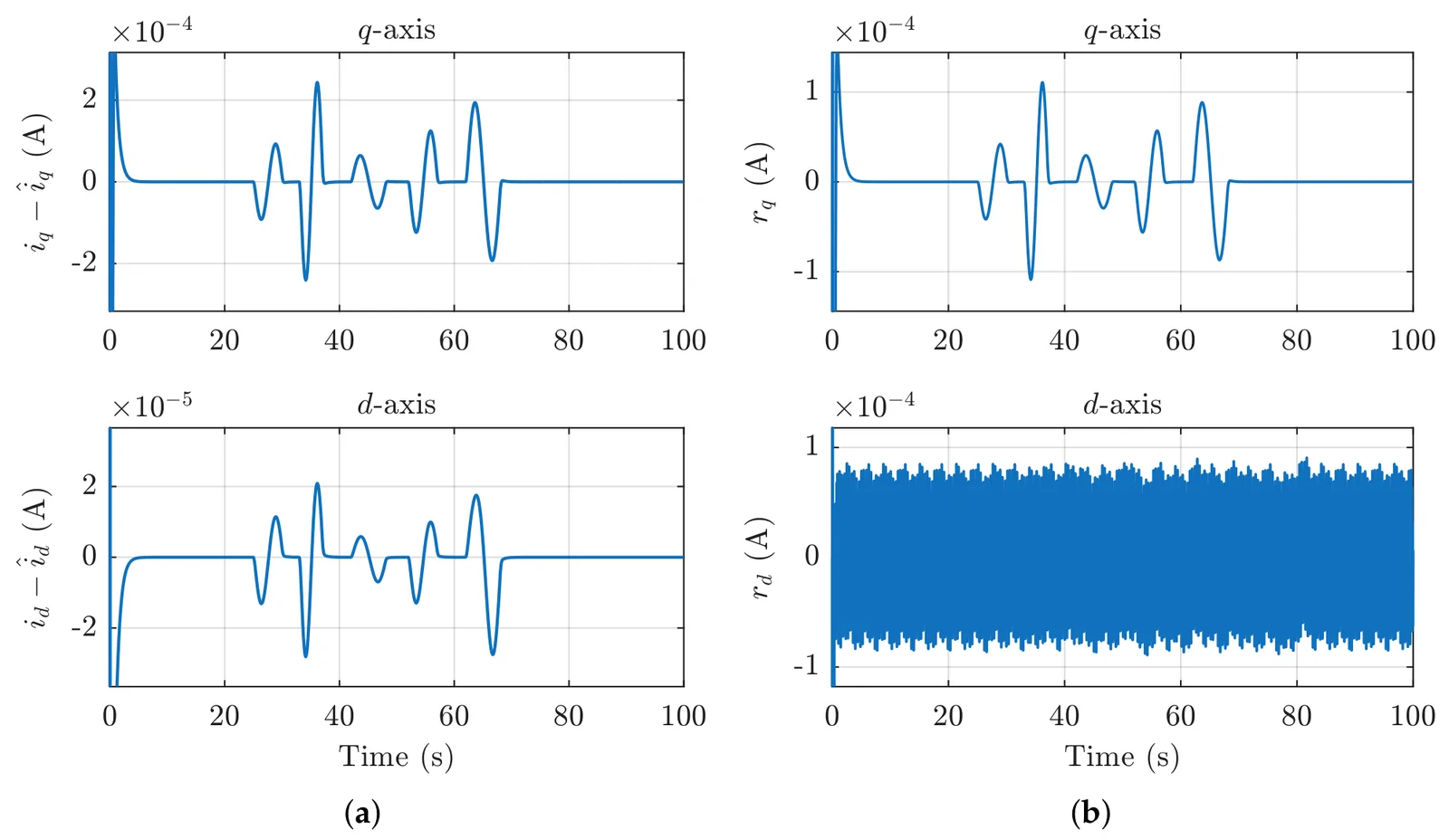
(**a**) Reconstruction error $Cx-C\hat{x}$; (**b**) output residual $r=Y-\hat{Y}$ ((**top**): *q*-axis; (**bottom**): *d*-axis; steady-state scale, initial transient truncated). The reconstruction error remains below $3\times 10^{-5}$ A on the *d*-axis and $3\times 10^{-4}$ A on the *q*-axis, and reacts only to the actuator-fault transitions; the residual $r_{d}$ contains only the ripple due to *w*.

**Figure 7:**
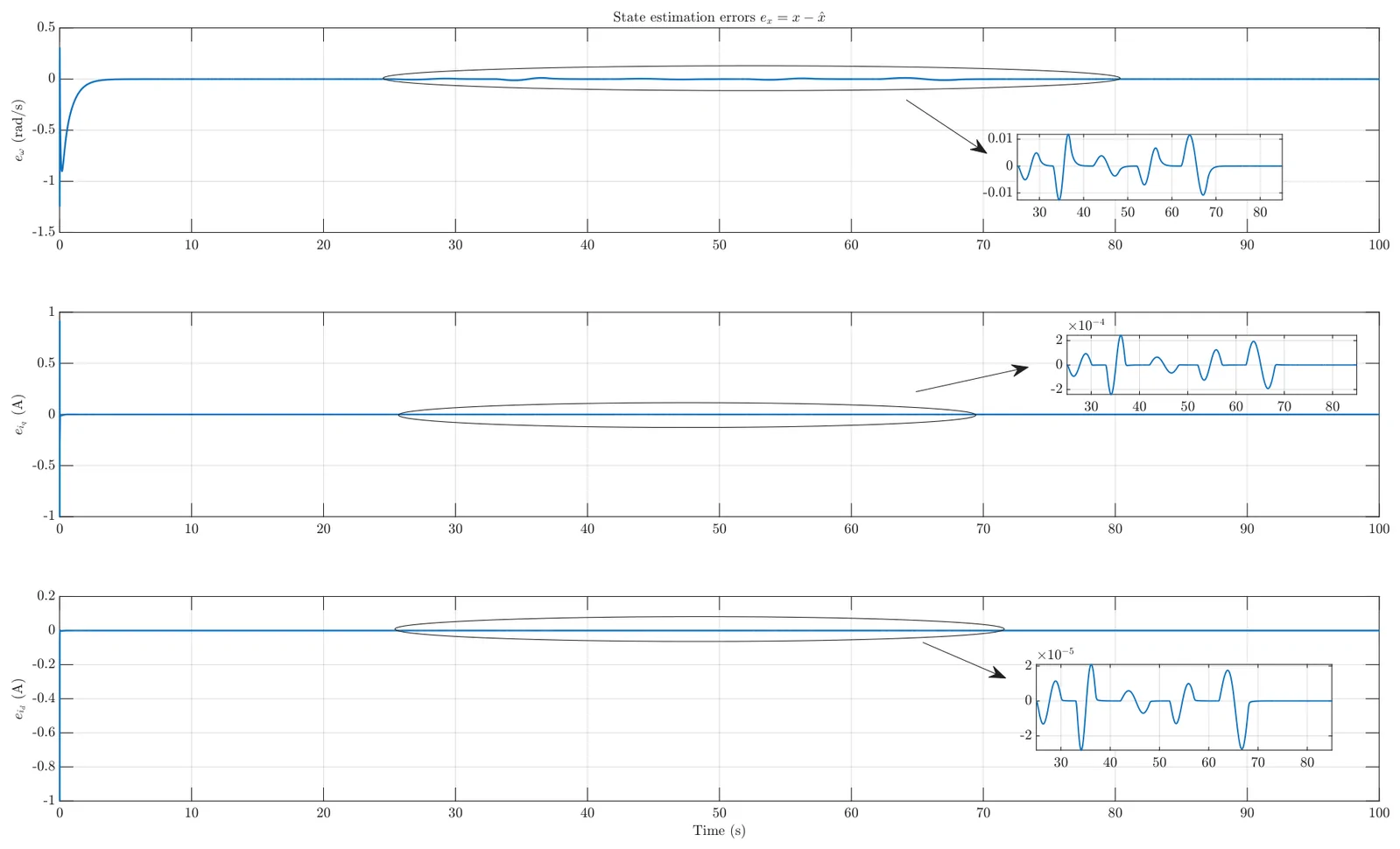
State estimation errors $e_{x}=x-\hat{x}$. The initial error caused by the erroneous observer initialization vanishes within a few seconds; the insets, over the fault injection windows, show that the errors remain bounded during both fault injection windows.

**Figure 8:**
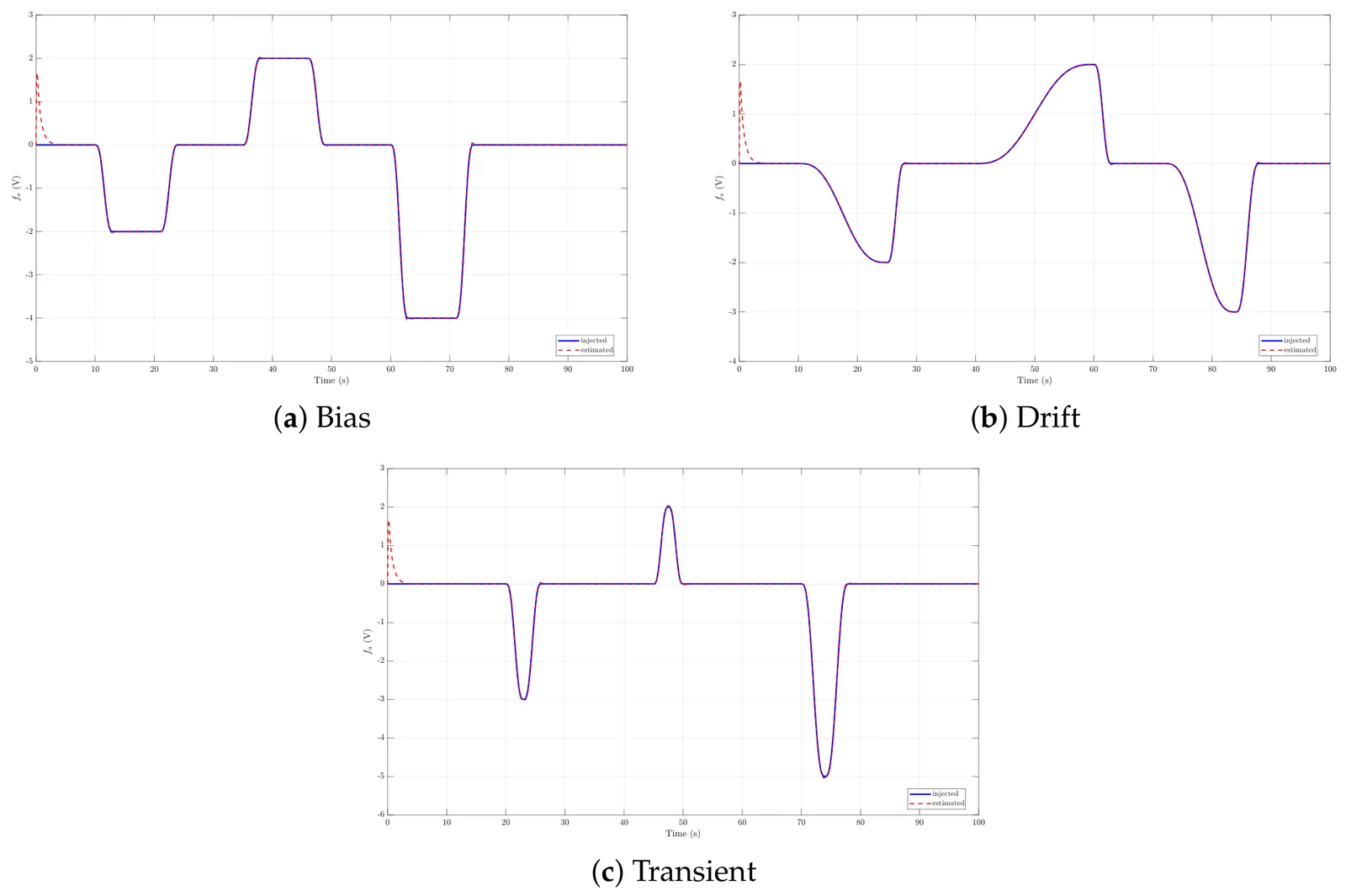
Reconstruction of the Scenario 2 actuator faults: (**a**) three biases, (**b**) three drifts, (**c**) three transients. Solid blue line: injected fault; dashed red line: estimated fault. In all three cases, the estimate tracks the injected profile and returns to zero between occurrences, with an RMSE of at most $3.2\times 10^{-2}$ V (Table 5).

**Figure 9:**
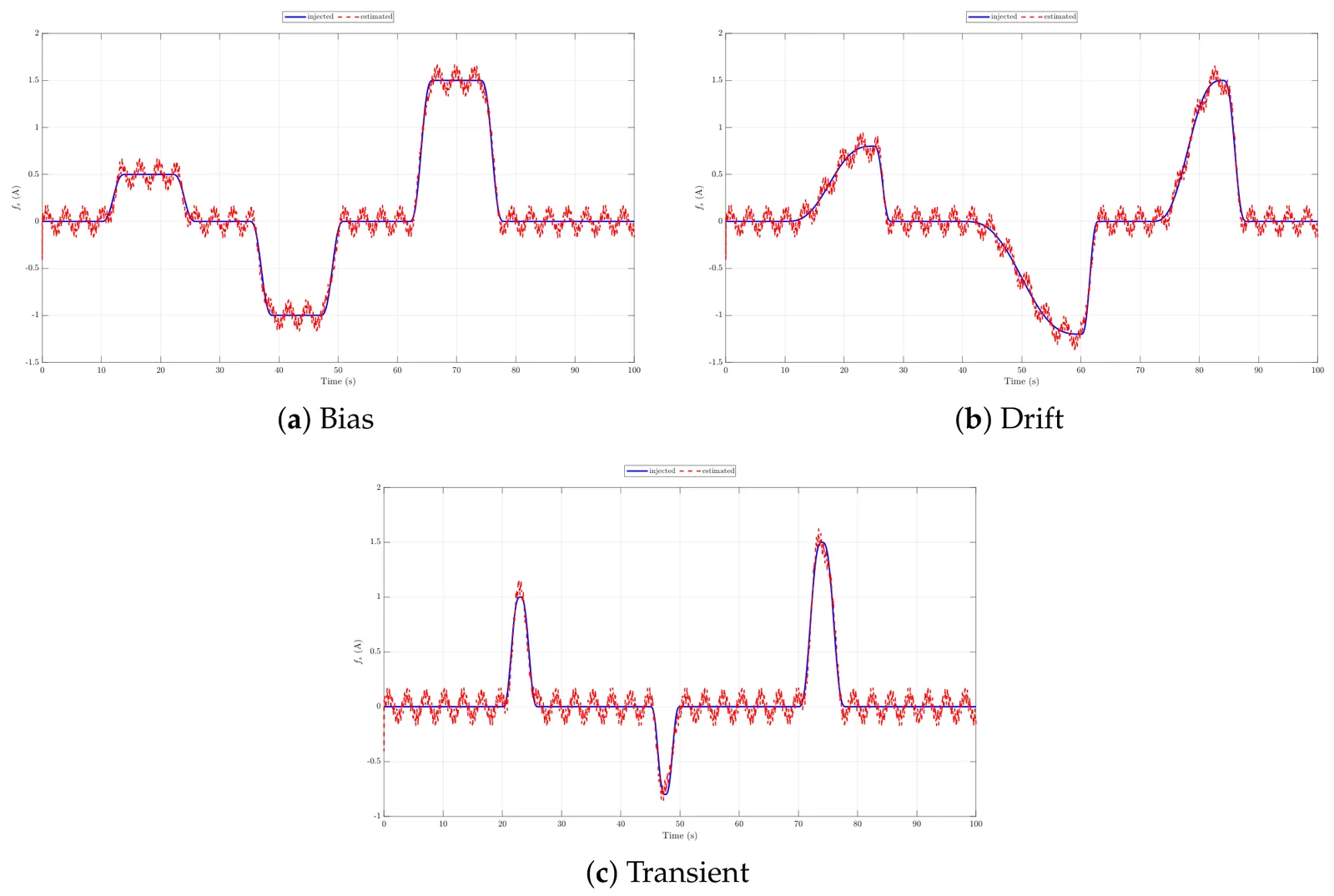
Reconstruction of the Scenario 3 sensor faults: (**a**) three biases, (**b**) three drifts, (**c**) three transients. Solid blue line: injected fault; dashed red line: estimated fault. The estimate tracks the injected profile up to the ±0.17 A ripple due to the disturbance acting on the same measurement channel (RMSE of $8.2\times 10^{-2}$ A, Table 5).

**Table 1:** Physical parameters of the PMSG and wind turbine model.

Parameter	Symbol	Value
Stator resistance	$R_{s}$	1.13Ω
Stator inductance	*L*	0.0027 H
Moment of inertia	*J*	0.005 kg·m^2^
Permanent magnet flux	$\phi_{mag}$	0.16 Wb
Number of pole pairs	$\rho_{n}$	10
Viscous friction coefficient	Λ	0.006 N·m·s/rad
Blade radius	R	0.5 m
Air density	ν	1.2 kg/m^3^
Maximum power coefficient	$C_{P_{\max}}$	0.441
Optimal tip-speed ratio	$\lambda_{opt}$	6.91

**Table 2:** Observer design parameters (common to the three scenarios) and Scenario 1 data.

Parameter	Symbol	Value
T–S operating range	$\left[d_{\min},d_{\max}\right]$	[0,10] rad/s
Filtering matrix	*E*	$10\;I_{2}$
Prescribed decay rate	β	0.7
Mismatch bounds	$\delta_{1}$, $\delta_{2}$	100, 0
Order/bound of the fault derivative (Scenario 1)	*k*, $f_{0}$	2, 0.982
Optimal attenuation level	$\mu^{*}$	1.875
Mismatch attenuation scalar	λ	$3\times 10^{4}$
Extreme eigenvalues of *P*	$\lambda_{\min}\left(P\right)$, $\lambda_{\max}\left(P\right)$	$1.27\times 10^{-3}$, $2.02\times 10^{5}$
LMI feasibility margin (largest eigenvalue, μ=1.9)	–	$-1.2\times 10^{-3}$
Rightmost eigenvalues at the vertices	maxReλ(Ai−KiC)	−1.25, −1.42
Bounds on $|\omega_{g}|$ and $∥{\left[\;i_{q}\;\;i_{d}\;\right]}^{T}∥$ (simulation)	$\rho_{\omega }$, $\rho_{i}$	7.16 rad/s, 8.86 A
Mismatch bound, ultimate bound (E=0)	$\bar{d}$, *b*	886, $3.6\times 10^{6}$
Initial observer estimate	$\hat{x}\left(0\right)$	${\left[1,\;1,\;1\right]}^{T}$

**Table 3:** Transitions $\left(t_{0},\;T_{r},\;M\right)$ of the Scenario 1 faults ($t_{0}$ and $T_{r}$ in seconds).

	1	2	3	4	5
$f_{a}$ (V)	(25,5,−1.5)	(33,4,−2.5)	(42,6,+1.5)	(52,5,−2.0)	(62,6,+4.5)
$f_{s}$ (A)	(40,5,+1.0)	(50,4,+1.5)	(58,6,−1.0)	(68,5,+0.8)	(76,6,−2.3)

**Table 4:** Quantitative performance indices of the proposed observer (Scenario 1, unmeasurable premise variable).

Index	Signal	Value
RMSE (states, t≥2 s)	$\omega_{g}$	$4.7\times 10^{-3}$ rad/s
	$i_{q}$	$5.6\times 10^{-5}$ A
	$i_{d}$	$6.1\times 10^{-6}$ A
RMSE (faults, injection window)	$f_{a}$ over [25,68] s	$8.7\times 10^{-3}$ V
	$f_{s}$ over [40,82] s	$8.2\times 10^{-2}$ A
5% convergence time	$\omega_{g}$	2.13 s
	$i_{q}$	0.017 s
	$i_{d}$	0.006 s
Reconstruction delay	$f_{a}$	<0.1 s
	$f_{s}$	within the disturbance ripple (±0.17 A)
Empirical $L_{2}$ gain	on $\tilde{e}$	0.58
	on $x-\hat{x}$	$1.8\times 10^{-2}$
Attenuation level (Theorem 1)	$\mu^{*}$	1.875

**Table 5:** Reconstruction indices of the six fault profiles (three occurrences per profile): Scenario 2 (actuator faults) and Scenario 3 (sensor faults). For sensor faults, the RMSE and the maximum error are dominated by the ±0.17 A ripple due to the disturbance acting on the same measurement channel.

Profile	RMSE	Max. Error	$f_{0}$	$\gamma_{\mathrm{emp}}$
Actuator bias	$1.9\times 10^{-2}$ V	$6.1\times 10^{-2}$ V	2.793	0.286
Actuator drift	$8.4\times 10^{-3}$ V	$3.0\times 10^{-2}$ V	1.396	0.473
Actuator transient	$3.2\times 10^{-2}$ V	$5.2\times 10^{-2}$ V	2.094	0.276
Sensor bias	$8.2\times 10^{-2}$ A	0.17 A	0.589	0.914
Sensor drift	$8.2\times 10^{-2}$ A	0.17 A	0.838	0.921
Sensor transient	$8.2\times 10^{-2}$ A	0.17 A	0.804	0.740

## Data Availability

The data presented in this study are generated by simulation from the models and parameters given in the article (Table 1 and Table 2, Appendix A).

## Appendix A. Numerical Observer Gains

The gains $K_{i}\in R^{9\times 2}$, which satisfy the conditions of Theorem 1 with $M_{i}=PK_{i}$ and the parameters of Table 2 are

$$K_{1}=10^{4}\;\left[\begin{array}{cc} 0.0836 & 0\\ 0.0014 & 0\\ 0.0002 & 0\\ 0.0012 & 0\\ -0.0023 & 0.0780\\ 0.1354 & 0\\ -0.0877 & 2.8047\\ 0.8659 & -0.0002\\ -0.1379 & 4.4095 \end{array}\right]\;,\;K_{2}=10^{4}\;\left[\begin{array}{cc} 0.0870 & 0\\ 0.0015 & 0\\ 0.0002 & 0\\ 0.0013 & 0\\ -0.0024 & 0.0780\\ 0.1409 & 0\\ -0.1005 & 2.8047\\ 0.9539 & -0.0002\\ -0.1580 & 4.4095 \end{array}\right]\;,$$

where rows 1–5 form $K_{P,i}$, rows 6 and 7 form $K_{I,i}$ and rows 8 and 9 form $K_{I,i}^{1}$ (entries displayed as 0 are smaller than $5\times 10^{-4}$ in absolute value).
